# Gender Disparities in Anti-dementia Medication Use among Older Adults: Health Equity Considerations and Management of Alzheimer's Disease and Related Dementias

**DOI:** 10.3389/fphar.2021.706762

**Published:** 2021-08-25

**Authors:** Z. Kevin Lu, Xiaomo Xiong, Xinyuan Wang, Jun Wu

**Affiliations:** ^1^ Department of Clinical Pharmacy and Outcomes Sciences, University of South Carolina, Columbia, SC, United States; ^2^ Key Laboratory of Cardiovascular and Cerebrovascular Medicine, School of Pharmacy, Nanjing Medical University, Nanjing, China; ^3^ Department of Pharmaceutical and Administrative Sciences, Presbyterian College School of Pharmacy, Clinton, SC, United States

**Keywords:** alzheimer’s disease and related diseases, ADRD, anti-dementia medications, gender disparities, medicare

## Abstract

**Objective:** The prevalence of Alzheimer’s disease and related dementias (ADRD) in women is higher than men. However, the knowledge of gender disparity in ADRD treatment is limited. Therefore, this study aimed to determine the gender disparities in the receipt of anti-dementia medications among Medicare beneficiaries with ADRD in the U.S.

**Methods:** We used data from the Medicare Current Beneficiary Survey 2016. Anti-dementia medications included cholinesterase inhibitors (ChEIs; including rivastigmine, donepezil, and galantamine) and N-methyl-D-aspartate (NMDA) receptor antagonists (including memantine). Descriptive analysis and multivariate logistic regression models were implemented to determine the possible gender disparities in the receipt of anti-dementia medications. Subgroup analyses were conducted to identify gender disparities among beneficiaries with Alzheimer’s disease (AD) and those with only AD-related dementias.

**Results:** Descriptive analyses showed there were statistically significant differences in age, marital status, and Charlson comorbidities index (CCI) between Medicare beneficiaries who received and who did not receive anti-dementia medications. After controlling for covariates, we found that female Medicare beneficiaries with ADRD were 1.7 times more likely to receive anti-dementia medications compared to their male counterparts (odds ratio [OR]: 1.71; 95% confidence interval [CI]: 1.19–2.45). Specifically, among Medicare beneficiaries with AD, females were 1.2 times more likely to receive anti-dementia medications (Odds Radio: 1.20; 95% confidence interval: 0.58–2.47), and among the Medicare beneficiaries with only AD-related dementias, females were 1.9 times more likely to receive anti-dementia medications (OR: 1.90; 95% CI: 1.23–2.95).

**Conclusion:** Healthcare providers should be aware of gender disparities in receiving anti-dementia medications among patients with ADRD, and the need to plan programs of care to support both women and men. Future approaches to finding barriers of prescribing, receiving and, adhering to anti-dementia medications by gender should include differences in longevity, biology, cognition, social roles, and environment.

## Background

Alzheimer’s disease and related dementias (ADRD) are typical neurodegenerative diseases, including Alzheimer’s disease (AD) and common AD-related dementias, such as Lewy body dementia, vascular dementia, and mild cognitive impairment (Galasko et al., 1994; Deb et al., 2017). ADRD are characterized by a decline in memory leading to loss of daily activities and the fifth leading cause of death among the adults aged 65 years or older in the U.S. (Galasko et al., 1994; Deb et al., 2017; Matthews et al., 2019). According to *2019 Alzheimer’s disease facts and figures*, the total number of Alzheimer’s patients was more than five million in 2019 and is expected to increase to 13.8 million by 2050 in the United States (US) (Alzheimer’s Association, 2019). The total annual costs of patients with ADRD are projected to increase to more than 1.1 trillion in 2050 in the US, with a fourfold increase in government spending under Medicare and Medicaid in the U.S (Alzheimer’s Association, 2019). Since there is still no treatment for curing ADRD currently, slowing the progression of ADRD is crucial to reducing the burden of patients (Hampel et al., 2018; McMichael et al., 2020).

Anti-dementia medications are a class of drugs used to slow the progression of ADRD (Anand et al., 2017; McMichael et al., 2020). To date, the anti-dementia medications approved by the US Food and Drug Administration (FDA) can be divided into two categories, including cholinesterase inhibitors (ChEIs; including rivastigmine, donepezil, and galantamine) and the N-methyl-D-aspartate (NMDA) receptor antagonists (including memantine) (Anand et al., 2017). Several reviews show that many randomized controlled trials (RCTs) have confirmed the effectiveness of these drugs in improving cognitive, behavioral problems, and neuropsychiatric symptoms (Birks, 2006; Winblad et al., 2007; van de Glind et al., 2013). A study also shows that persistent treatment with anti-dementia medications can slow the clinical progression of ADRD (Rountree et al., 2009). The clinical guidelines for anti-dementia medications recommend that cholinesterase inhibitors are effective for mild to moderate ADRD, while memantine is helpful for moderate to severe ADRD (O'Brien et al., 2017).

Gender disparities have always been an unresolved issue in patients with ADRD. Evidence has documented differences between men and women in terms of brain structure and function over the lifespan (Azad et al., 2007; Cosgrove et al., 2007; Mielke et al., 2014), and proposed some part of the mechanism for explaining the gender imbalance in ADRD, including biological explanation (genetics, hormones) and social explanation (education, occupation, cognitive activity) (Azad et al., 2007; Cosgrove et al., 2007; Mielke et al., 2014). Also, studies show that the prevalence of women with ADRD is significantly higher than that of men (Winblad et al., 2016; Alzheimer’s Disease International, 2015). Medicare is a federal health insurance program for people who are 65 or older, certain younger people with disabilities, and people with End-Stage Renal disease (Centers for Medicare and Medicaid Services, 2021a). Given that females constitute the majority of Medicare beneficiaries (National Committee to Preserve Social Security and Medicare, 2021), and that anti-dementia drugs are perennially in the top 15 therapeutic classes of drugs covered under Part D (Koller et al., 2016), it’s critical to understand the gender disparities in the receipt of anti-dementia medications among Medicare beneficiaries with ADRD. However, to date, little evidence has investigated the gender disparities among the beneficiaries with ADRD.

This study used a nationally representative database to determine the gender disparities in the receipt of anti-dementia medications among Medicare beneficiaries with ADRD. Given that evidence has shown that females are more likely to use preventive care services compared to males (Vaidya et al., 2012; Owens, 2018), we hypothesized that compared to male Medicare beneficiaries with ADRD, those female beneficiaries are more likely to receive anti-dementia medications.

## Methods

### Study Design and Data Source

We conducted a retrospective cross-sectional study to determine gender disparities in the receipt of anti-dementia medications among Medicare beneficiaries. Data were derived from the Medicare Current Beneficiary Survey (MCBS) in 2016. MCBS is a nationally comprehensive and authoritative survey of Medicare beneficiaries, which is sponsored by the Centers for Medicare and Medicare Services (CMS) (Adler, 1994; Centers for Medicare and Medicaid Services, 2021b). The core purpose of MCBS is to help CMS manage the Medicare program and understand the health and welfare of beneficiaries (Adler, 1994; Centers for Medicare and Medicaid Services, 2021b). The MCBS sample is selected from Medicare Administrative Enrollment (MAE) data using a rotating panel design that tracks each beneficiary up to 4 years with 12 interviews (Adler, 1994; Centers for Medicare and Medicaid Services, 2021b). When participants are no longer able to conduct in-person interviews due to unconsciousness, they can name proxy respondents to answer survey questions on their behalf. MCBS releases three data set annually, which collect a wealth of information about Medicare beneficiaries’ demographic characteristics, insurance, health status, and the usage and cost of all medical services (Adler, 1994; Centers for Medicare and Medicaid Services, 2021b). Then the information is merged with Medicare part A and B claims and finally formed a continuous, multi-purpose survey (Adler, 1994; Centers for Medicare and Medicaid Services, 2021b).

### Study Population

Prevalent patients with ADRD aged 65 years or older were identified if any of their claims between January 1, 2016, through December 31, 2016 included the International Classification of Diseases, 10th Revision, Clinical Modification (ICD-10-CM) codes for AD and AD-related dementias. Specifically, the ICD-10 CM codes for AD was G30. AD-related dementias included Lewy-body associated dementia (G31.83), mild cognitive impairment (G31.84), frontotemporal dementia (G31.0), vascular dementia (F01), and non-specific dementias (G31.1, F00, F02, F03, F05.1). These codes were derived from the chronic conditions data warehouse algorithms (Gorina and Kramarow, 2011).

We excluded participants who were eligible for the Medicare program due to end-stage renal disease (ESRD) or disability, and those who joined the Health Maintenance Organization (HMO) during the study period.

### Measures

Anti-dementia medications were identified based on the U.S. FDA’s prescription database (U.S. Food and Drug Administration, 2021). The FDA’s prescription database covers all drugs that have been approved by the FDA to be marketed in the US cholinesterase inhibitors included rivastigmine, donepezil, galantamine, and memantine is the only available NMDA receptor antagonist (Rountree et al., 2009; U.S. Food and Drug Administration, 2021). MCBS participants who received anti-dementia medications were identified if they had at least one prescription of cholinesterase inhibitors and the NMDA receptor antagonists in the study period.

Covariates were based on previous research on similar topics using MCBS (Bhattacharjee et al., 2012; Brown-Guion et al., 2013; Lee and Khan, 2016). We extracted demographic, socioeconomics, and health-related factors used as covariates. Demographic factors included age (≥65 and <75, ≥75 and <85, and ≥85), race (Hispanic Caucasian, Non-Hispanic African American, Hispanic, and others), marital status (married and non-married), living county (non-Rural, micropolitan, and metropolitan), census region (Northeast, North central/Midwest, South, and West), and educational attainment (<High school graduate, high school graduate, > high school graduate). Socioeconomic factors included income (<$25,000, ≥ $25,000 and < $50,000, ≥ $50,000 and < $75,000, and ≥ $75,000). Health-related factors included Charlson comorbidity index (CCI; 0, 1, 2, ≥3). Specifically, both demographic and socioeconomic factors were identified based on the MCBS’s survey data, while the CCI was calculated based on Medicare claims (Formiga et al., 2009).

### Statistical Analysis

Descriptive analysis was used to compare the difference in the characteristics between users and non-users of anti-dementia medications among Medicare beneficiaries with ADRD. Chi-square tests were used to compare the difference in the categorical variables. Multivariate logistic regression models were used to estimate the association between gender and the receipt of anti-dementia medications after controlling for covariates. We also conducted two subgroup analyses, one for beneficiaries with AD and the other for those with only AD-related dementias. SAS software (version 9.4; Statistical Analysis Systems, NC, USA) was used to perform the statistical analyses, and the level of statistical significance was set at *p* < 0.05.

## Results

**FIGURE 1:**
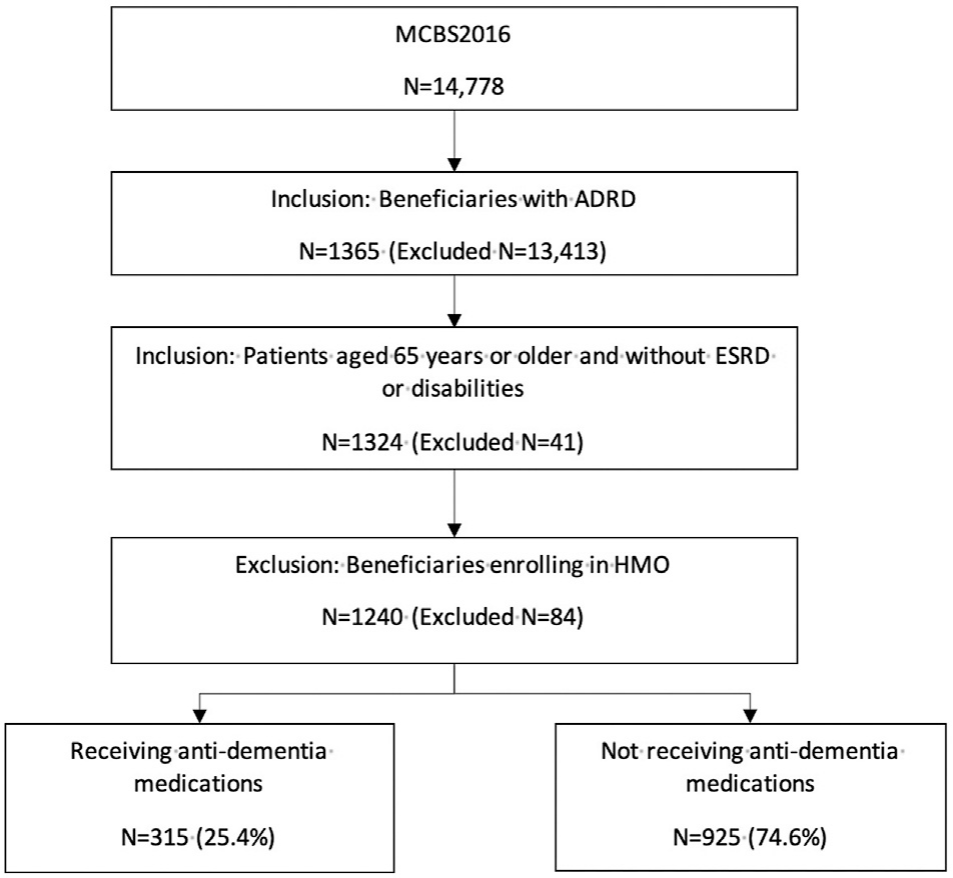
Flowchart of study population selection.

**FIGURE 2:**
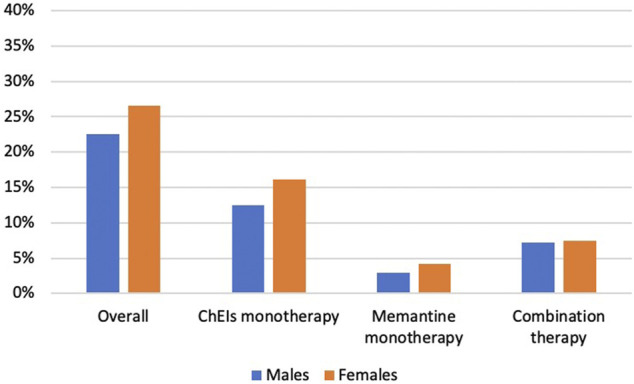
Proportion of the receipt of different types of anti-dementia medications by gender. ADRD: Alzheimer’s diseases and related dementias; AD: Alzheimer’s disease; ChEIs: cholinesterase inhibitors.

**FIGURE 3:**
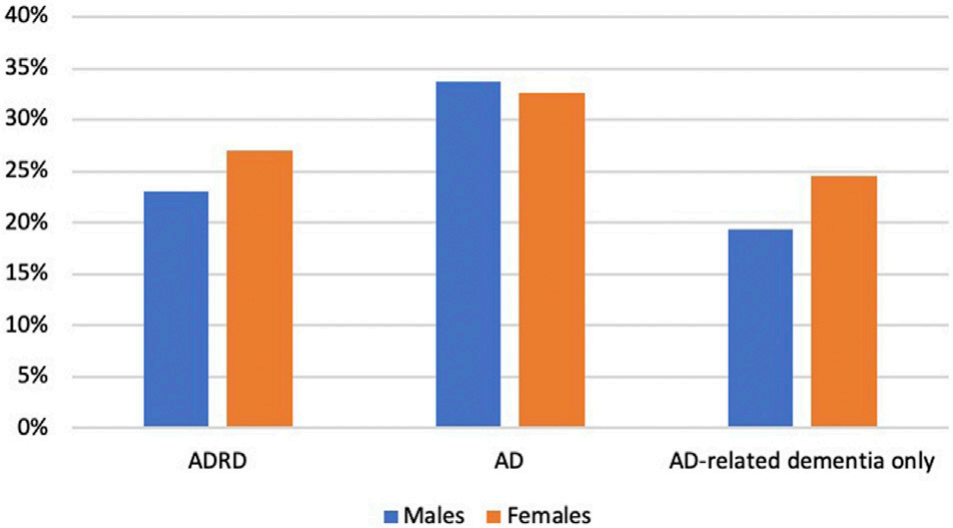
Proportion of the receipt of different types of anti-dementia medications by types of ADRD: Alzheimer’s diseases and related dementias; AD: Alzheimer’s disease; ChEIs: cholinesterase inhibitors.

1,240 Medicare beneficiaries with ADRD were identified out of 14,778 beneficiaries in MCBS 2016 based on the inclusion and exclusion criteria (Figure 1). Among the beneficiaries with ADRD, 315 (25.4%) received anti-dementia medications, and 925 (74.6%) did not receive. A total of 85 (22.6%) males received anti-dementia medications, compared with 230 (26.6%) in females. Specifically, the proportion of the receipt of ChEIs monotherapy was 12.5% among males and 16.1% among females, the proportion of the receipt of memantine monotherapy was 2.9% among males and 4.2% among females. The proportion of combination therapy was 7.2% among males and 7.4% among females (Figure 2). In addition, 29 out of 86 (33.7%) male patients with AD received anti-dementia medications, while it was 72 out of 221 (32.6%) among females. Meanwhile, 56 out of 290 (19.3%) male patients with AD-related dementias received the medications, and it was 158 out of 643 (24.6%) among female patients (Figure 3). According to Table 1, Compared to those who did not receive anti-dementia medications, Medicare beneficiaries with ADRD who received the medication were more likely to be aged between 75 and 85 years (*p* = 0.006), not married (*p* = 0.032), and have fewer comorbidities (*p* = 0.007).

**TABLE 1:** Base-line demographics and comparison.

Factors	Non-users	%	Users	%	*p*-value
	N = 925, no		N = 315, no		
**Gender**					0.136
Male	291	31.5	85	27.0	
Female	634	68.5	230	73.0	
**Age**					0.006
≥65 and <75	91	9.8	31	9.8	
≥75 and <85	273	29.5	123	39.0	
≥85	561	60.6	161	51.1	
**Marital status**					0.032
Married	658	71.9	205	65.5	
Non married	257	28.1	108	34.5	
**Race**					0.535
Non-Hispanic Caucasian	777	86.6	267	87.8	
Non-Hispanic African American	90	10.0	24	7.9	
Hispanic	30	3.3	13	4.3	
Others	35	3.9	31	10.2	
**Living county**					0.146
Rural	65	7.0	17	5.4	
Micropolitan	138	14.9	61	19.4	
Metropolitan	722	78.1	237	75.2	
**Census Region**					0.094
Northeast	178	19.3	63	20.0	
North Central/Midwest	210	22.8	83	26.3	
South	380	41.2	134	42.5	
West	155	16.8	35	11.1	
**Education**					0.379
< High school graduate	176	24.9	77	29.2	
High school graduate	363	51.4	131	49.6	
> High school graduate	167	23.7	56	21.2	
**Family income**					0.374
< $25,000	584	67.0	186	62.4	
≥ $25,000 and < $50,000	153	17.6	65	21.8	
≥ $50,000 and < $75,000	92	10.6	34	11.4	
≥ $75,000	42	4.8	13	4.4	
**CCI**					0.007
0	135	14.6	50	15.9	
1	251	27.1	115	36.5	
2	197	21.3	57	18.1	
≥3	342	37.0	93	29.5	

CCI: Charlson comorbidity index.

After controlling for covariates, female Medicare beneficiaries with ADRD were 1.7 times more likely to receive anti-dementia medications compared to males (odds ratio [OR]: 1.71; 95% confidence interval [CI]: 1.19–2.45; Table 2). 307 and 933 Medicare beneficiaries with AD and AD-related dementias only were identified for subgroup analysis, respectively. According to Table 3, Among Medicare beneficiaries with AD, compared to males, females were 1.2 times more likely to receive anti-dementia medications, however, it’s not statistically significant (OR: 1.20; 95% CI: 0.58–2.47). Based on Table 4, among the group of Medicare beneficiaries with only AD-related dementias, compared to males, females were 1.9 times more likely to receive anti-dementia medications (OR: 1.90; 95% CI: 1.23–2.95). The goodness of fit of each regression model can be found in Supplementary Material.

**TABLE 2:** Results of logistic regression among beneficiaries with ADRD (N = 1,240).

Variables	OR	Lower limit	Upper limit
**Gender**			
Male	Ref		
Female	1.71	1.19	2.45
**Age**			
≥65 and <75	Ref		
≥75 and <85	1.29	0.76	2.21
≥85	0.77	0.45	1.30
**Marital status**			
Married	Ref		
Non married	1.27	0.88	1.82
**Race**			
Non-Hispanic Caucasian	Ref		
Non-Hispanic African American	0.77	0.44	1.36
Hispanic	1.35	0.61	2.99
Others	2.13	0.91	5.03
**Living county**			
Rural	Ref		
Micropolitan	1.64	0.82	3.27
Metropolitan	1.36	0.72	2.55
**Census region**			
Northeast	Ref		
North Central/Midwest	1.20	0.76	1.90
South	0.99	0.64	1.51
West	0.69	0.39	1.22
**Education**			
< High school graduate	Ref		
High school graduate	0.79	0.54	1.15
> High school graduate	0.62	0.38	1.02
**Family income**			
< $25,000	Ref		
≥ $25,000 and < $50,000	1.41	0.95	2.11
≥ $50,000 and < $75,000	1.23	0.75	2.02
≥ $75,000	1.04	0.47	2.30
**CCI**			
0	Ref		
1	1.37	0.87	2.15
2	0.70	0.41	1.17
≥3	0.65	0.41	1.04

The ref group represents the row in the variable column.

ADRD: Alzheimer’s disease and related dementias; CCI: Charlson comorbidity index; OR: Odds ratio; Ref: Reference.

**TABLE 3:** Results of logistic regression among beneficiaries with AD (N = 307).

Variables	OR	Lower limit	Upper limit
**Gender**			
Male	Ref		
Female	1.20	0.58	2.47
**Age**			
≥65 and <75	Ref		
≥75 and <85	1.18	0.37	3.77
≥85	0.48	0.15	1.55
**Marital status**			
Married	Ref		
Non married	0.82	0.39	1.72
**Race**			
Caucasian	Ref		
African American	0.88	0.33	2.35
Hispanic	0.98	0.39	2.45
Asian	0.77	0.25	2.42
**Living county**			
Rural	Ref		
Micropolitan	5.55	1.11	27.62
Metropolitan	2.74	0.62	12.19
**Census region**			
Northeast	Ref		
North Central/Midwest	0.88	0.33	2.35
South	0.98	0.39	2.45
West	0.77	0.25	2.42
**Education**			
< High school graduate	Ref		
High school graduate	0.72	0.32	1.59
> High school graduate	0.69	0.25	1.95
**Family income**			
< $25,000	Ref		
≥ $25,000 and < $50,000	1.98	0.81	4.83
≥ $50,000 and < $75,000	1.44	0.54	3.86
≥ $75,000	1.01	0.20	5.14
**CCI**			
0	Ref		
1	2.38	0.95	5.96
2	0.79	0.28	2.26
≥3	0.62	0.23	1.65

The ref group represents the row in the variable column.

AD: Alzheimer’s disease; CCI: Charlson comorbidity index; OR: Odds ratio; Ref: Reference.

**TABLE 4:** Results of logistic regression among beneficiaries with AD-related dementias (N = 993).

Variables	OR	Lower limit	Upper limit
**Gender**			
Male	Ref		
Female	1.90	1.23	2.95
**Age**			
≥65 and <75	Ref		
≥75 and <85	1.20	0.64	2.24
≥85	0.85	0.46	1.57
**Marital status**			
Married	Ref		
Non married	1.42	0.92	2.20
**Race**			
Caucasian	Ref		
African American	0.72	0.37	1.40
Hispanic	0.76	0.26	2.22
Asian	1.94	0.72	5.20
**Living county**			
Rural	Ref		
Micropolitan	1.34	0.61	2.96
Metropolitan	1.21	0.59	2.46
**Census region**			
Northeast	Ref		
North Central/Midwest	1.30	0.77	2.22
South	0.95	0.58	1.56
West	0.57	0.28	1.15
**Education**			
< High school graduate	Ref		
High school graduate	0.75	0.48	1.17
> High school graduate	0.56	0.31	1.02
**Family income**			
< $25,000	Ref		
≥ $25,000 and < $50,000	1.33	0.83	2.12
≥ $50,000 and < $75,000	1.16	0.63	2.12
≥ $75,000	1.16	0.46	2.97
**CCI**			
0	Ref		
1	1.14	0.66	1.96
2	0.60	0.32	1.13
≥3	0.65	0.37	1.12

The ref group represents the row in the variable column.

AD: Alzheimer’s disease; CCI: Charlson comorbidity index; OR: Odds ratio; Ref: Reference.

## Discussion

This study analyzed nationally representative data on 1,240 Medicare beneficiaries with ADRD aged 65 years or older to investigate gender disparities in the receipt of anti-dementia medications. Our study found gender disparities in the receipt of anti-dementia medications among Medicare beneficiaries with ADRD. Specifically, female Medicare beneficiaries with ADRD are 1.7 times more likely to receive anti-dementia medications compared to their male counterparts.

The overall proportion of the receipt of anti-dementia medications was 26.1%, which was consistent with a previous study using Medicare claims that found 26% of patients with ADRD receiving at least one anti-dementia medication (Zuckerman et al., 2008). However, compared to some studies conducted in countries other than the US, the prevalence of using anti-dementia medications in our study is relatively low. A cross-sectional study in Finland found that 69% of patients in home care and residential care with dementia used anti-dementia medications (Kuronen et al., 2015). In a registry-based study in Sweden, more than 80% of AD patients used anti-dementia drugs, and in another study using registry in Spain, more than 50% of dementia patients were found receiving anti-dementia medications (Johnell et al., 2008; Avila-Castells et al., 2013). The reasons for such low prescribing rates in the United States are unclear and might require future research to investigate.

Our results do not explain why female Medicare beneficiaries with ADRD are more likely to receive anti-dementia medications. We posit that this could reflect the notion that the prevalence of ADRD is higher in women and that women are at a greater risk compared to men (Mielke et al., 2014). About two-thirds of patients diagnosed with AD dementia are women, and healthcare providers focus too much on female patients with ADRD despite evidence showing that there is no sex or gender difference in risk factors or mechanisms (Mazure and Swendsen, 2016). In addition, over the past few decades, the improvement in education and careers in women may have led to female patients being more willing to receive primary care and medications (Matthews et al., 2013; Langa et al., 2017). An article has demonstrated that women tend to use more services and spend more health care costs than men, and men often have insufficient awareness of medical treatment (Owens, 2018). It is also important to note that men and women may respond differently to anti-dementia medications, some systematic reviews have confirmed the importance of controlling the symptoms of patients with ADRD (Fox et al., 2012; Matsunaga et al., 2014). Evidence from observational studies has shown that in the treatment of donepezil and rivastigmine, the response of female patients is significantly better than that of male patients (Scacchi et al., 2014). The perception of a higher benefit from anti-dementia medications in female patients compared with male patients with ADRD may determine the gender disparities in receiving anti-dementia medications.

Although we found significant gender disparities in the receipt of anti-dementia medications among patients with ADRD and those with only AD-related dementia, there were no significant disparities among patients with AD. Among patients with AD, the proportion of males and females receiving anti-dementia medications was 33.7 and 32.6%, respectively. Meanwhile, the proportion of patients with AD receiving anti-dementia medication was higher than that of patients with only AD-related dementia. Patients with AD were more likely to be recommended for medications by their physicians than those with only AD-related dementias only. In this case, the gender difference might be reduced. In addition, there were no gender disparities in the receipt of anti-dementia medication before controlling for covariates. However, although we found no significant association between receipt of anti-dementia medication and any covariate, gender disparities emerged after controlling for covariates using multivariate logistic regression, implying that some covariates may have an impact on the receipt of antidementia medications. Future research should focus on identifying influential predictors of receipt of anti-dementia medications among patients with ADRD.

Gender disparities combine environmental, social, and cultural differences between women and men (Institute of Medicine (US) et al., 2001), which indicates that the gender disparities in ADRD are not only on biological factors, but also on education, nursing, and psychological health (Nebel et al., 2018). More and more evidence has indicated that sex and gender will affect the etiology, performance, and treatment results of many diseases. Compared with other medical fields such as cardiovascular disease, research on gender differences in ADRD is still in its infancy. Salim S. Virani et al. have found that a better understanding of gender differences can improve the care and treatment of patients with cardiovascular disease (Virani et al., 2015). We speculate that the same positive results may occur for patients with ADRD.

To our knowledge, this study is the first research to determine the gender disparities of Medicare beneficiaries in receiving anti-dementia medications. The anti-dementia medications included in our study are in line with the recommendation of the latest available treatment guideline for AD in the US (Winslow et al., 2011). Our study can provide important information for ADRD patients, healthcare providers, and policymakers in future clinical practice. First, special attention by healthcare providers or family caregivers should be given to male patients with ADRD and increase the use of anti-dementia drugs, as they are more likely to ignore their health status and spend less money on healthcare compared with their female counterparts (Langa et al., 2017). In future clinical practice, strategies such as increasing home care providers, education by emphasizing the importance of being adherent to anti-dementia drugs for male patients can be considered. Secondly, our findings from this study warrant the need to plan programs of care to support both women and men living with ADRD, their families, and their communities. Also, identifying where there are differences provides the potential for better treatment and care for both women and men. Finally, observational studies on the response of men and women to anti-dementia drugs are still controversial, Gallucci M (Gallucci et al., 2016) and Wattmo C’s (Wattmo et al., 2011) studies have pointed out that the response to cholinesterase inhibitors treatment and longitudinal cognitive outcomes were better in males, while Haywood WM et al. indicated there is no significant sex difference (Haywood and Mukaetova-Ladinska, 2006). On the other hand, there is no data on sex-related pharmacokinetic anti-dementia medications. Overall, whether and how gender affects the effectiveness and safety of anti-dementia medications needs further studies and we should increase efforts to collect data on gender disparities, such as in post-marketing surveillance studies (Clerici et al., 2012).

However, this study also has several limitations. First, in order to achieve a sample size that is sufficient to detect the effect size, we did not include some health-related covariates with too many missing values, such as body mass index (BMI), difficulty in activities of daily living (ADL), and instrumental activities of daily living (IADL). Second, this study was unable to measure the disease severity of cognitive impairment due to a lack of related information, which may influence anti-dementia drug use. Third, the results of this study only reflected the prescribing patterns of anti-dementia medication in older patients covered by Medicare in the US. The conclusion might not be generalized to other US populations covered by other public or private insurance plans and populations in other countries. Finally, due to the cross-sectional design, we could not draw a causal conclusion on the receipt of anti-dementia medication between male and female Medicare beneficiaries with ADRD.

## Conclusion

In conclusion, our study found that gender disparities exist in the receipt of anti-dementia medications among Medicare beneficiaries with ADRD. Gender is rooted in biology, but it is primarily shaped by environment and experience. Healthcare providers should understand the gender disparities in receipt of anti-dementia drugs and provide interventions to improve prescribing patterns and patient adherence, not only on biological gender, but also on education, nursing, and psychological health factors.

## Data Availability

The data analyzed in this study is subject to the following licenses/restrictions: MCBS is Available for purchase from CMS after execution of an approved data use agreement. Requests to access these datasets should be directed to https://www.cms.gov/research-statistics-data-and-systems/research/mcbs.

## References

[B1] Adler G. S. (1994). A Profile of the Medicare Current Beneficiary Survey. Health Care Financ. Rev. 15, 153–163. 10138483 PMC4193434

[B2] Alzheimer's Association (2019). Alzheimer's Disease Facts and Figures. Alzheimer's Demen. 3, 321–387. 10.1016/j.jalz.2019.01.010

[B3] Alzheimer's Disease International (2015). Women and Dementia: A Global Research Review. Available online: https://www.alz.co.uk/women-and-dementia (Accessed Mar 6, 2021).

[B4] Anand A. Patience A. A. Sharma N. Khurana N. (2017). The Present and Future of Pharmacotherapy of Alzheimer's Disease: A Comprehensive Review. Eur. J. Pharmacol. 815, 364–375. 10.1016/j.ejphar.2017.09.043 28978455

[B5] Avila-Castells P. Garre-Olmo J. Calvó-Perxas L. Turró-Garriga O. Alsina E. Carmona O. (2013). Drug Use in Patients with Dementia: a Register-Based Study in the Health Region of Girona (Catalonia/Spain). Eur. J. Clin. Pharmacol. 69 (5), 1047–1056. 10.1007/s00228-012-1451-y 23179177

[B6] Azad N. A. Al Bugami M. Loy-English I. (2007). Gender Differences in Dementia Risk Factors. Gend. Med. 4, 120–129. 10.1016/s1550-8579(07)80026-x 17707846

[B7] Bhattacharjee S. Findley P. A. Sambamoorthi U. (2012). Understanding Gender Differences in Statin Use Among Elderly Medicare Beneficiaries: an Application of Decomposition Technique. Drugs Aging 29 (12), 971–980. 10.1007/s40266-012-0032-1 23160960

[B8] Birks J. (2006). Cholinesterase Inhibitors for Alzheimer's Disease. Cochrane Database Syst. Rev. 1, CD005593. 10.1002/14651858.CD005593 PMC9006343 16437532

[B9] Brown-Guion S. Y. Youngerman S. M. Hernandez-Tejada M. A. Dismuke C. E. Egede L. E. (2013). Racial/ethnic, Regional, and Rural/urban Differences in Receipt of Diabetes Education. Diabetes Educ. 39 (3), 327–334. 10.1177/0145721713480002 23482514

[B10] Centers for Medicare and Medicaid Services (2021a). What's Medicare? Available at: https://www.medicare.gov/what-medicare-covers/your-medicare-coverage-choices/whats-medicare (Accessed August 10, 2021).

[B11] Centers for Medicare and Medicaid Services (2021b). Medicare Current Beneficiary Survey (MCBS). Available online: https://www.cms.gov/Research-Statistics-Data-and-Systems/Research/MCBS (Accessed Mar 6, 2021).

[B12] Clerici F. Vanacore N. Elia A. Spila-Alegiani S. Pomati S. Da Cas R. (2012). Memantine Effects on Behaviour in Moderately Severe to Severe Alzheimer's Disease: A Post-Marketing Surveillance Study. Neurol. Sci. 33, 23–31. 10.1007/s10072-011-0618-0 21584738

[B13] Cosgrove K. P. Mazure C. M. Staley J. K. (2007). Evolving Knowledge of Sex Differences in Brain Structure, Function, and Chemistry. Biol. Psychiatry 62, 847–855. 10.1016/j.biopsych.2007.03.001 17544382 PMC2711771

[B14] Deb A. Thornton J. D. Sambamoorthi U. Innes K. (2017). Direct and Indirect Cost of Managing Alzheimer's Disease and Related Dementias in the United States. Expert Rev. Pharmacoecon Outcomes Res. 17 (2), 189–202. 10.1080/14737167.2017.1313118 28351177 PMC5494694

[B15] FormigaF.FortI.RoblesM. J.RiuS.SabartesO.BarrancoE. (2009). Comorbidity and Clinical Features in Elderly Patients with Dementia: Differences According to Dementia Severity. J. Nutr. Health Aging 13, 423–427. 10.1007/s12603-009-0078-x 19390748

[B16] Fox C. Crugel M. Maidment I. Auestad B. H. Coulton S. Treloar A. (2012). Efficacy of Memantine for Agitation in Alzheimer's Dementia: A Randomised Double-Blind Placebo Controlled Trial. PLoS One 7, e35185. 10.1371/journal.pone.0035185 22567095 PMC3342281

[B17] Galasko D. Hansen L. A. Katzman R. Wiederholt W. Masliah E. Terry R. (1994). Clinical-Neuropathological Correlations in Alzheimer's Disease and Related Dementias. Arch. Neurol. 51, 888–895. 10.1001/archneur.1994.00540210060013 8080388

[B18] Gallucci M. Spagnolo P. Aricò M. Grossi E. (2016). Predictors of Response to Cholinesterase Inhibitors Treatment of Alzheimer's Disease: Date Mining from the TREDEM Registry. J. Alzheimers Dis. 50, 969–979. 10.3233/JAD-150747 26836164

[B19] Gorina Y. Kramarow E. A. (2011). Identifying Chronic Conditions in Medicare Claims Data: Evaluating the Chronic Condition Data Warehouse Algorithm. Health Serv. Res. 46, 1610–1627. 10.1111/j.1475-6773.2011.01277.x 21649659 PMC3207195

[B20] Hampel H. Mesulam M. M. Cuello A. C. Farlow M. R. Giacobini E. Grossberg G. T. (2018). The Cholinergic System in the Pathophysiology and Treatment of Alzheimer's Disease. Brain 141, 1917–1933. 10.1093/brain/awy132 29850777 PMC6022632

[B21] Haywood W. M. Mukaetova-Ladinska E. B. (2006). Sex Influences on Cholinesterase Inhibitor Treatment in Elderly Individuals with Alzheimer's Disease. Am. J. Geriatr. Pharmacother. 4, 273–286. 10.1016/j.amjopharm.2006.09.009 17062329

[B22] Institute of Medicine (US) (2001). “Committee on Understanding the Biology of Sex and Gender Differences,” in Exploring the Biological Contributions to Human Health: Does Sex Matter?. Editors Wizemann T. M. Pardue M. L. (Washington (DC): National Academies Press (US)). 25057540

[B23] Johnell K. Weitoft G. R. Fastbom J. (2008). Education and Use of Dementia Drugs: A Register-Based Study of over 600,000 Older People. Dement Geriatr. Cogn. Disord. 25 (1), 54–59. 10.1159/000111534 18037814

[B24] Koller D. Hua T. Bynum J. P. (2016). Treatment Patterns with Antidementia Drugs in the United States: Medicare Cohort Study. J. Am. Geriatr. Soc. 64, 1540–1548. 10.1111/jgs.14226 27341454 PMC5045869

[B25] Kuronen M. Koponen H. Nykänen I. Karppi P. Hartikainen S. (2015). Use of Anti-Dementia Drugs in Home Care and Residential Care and Associations with Neuropsychiatric Symptoms: A Cross-Sectional Study. BMC Geriatr. 15, 100. Published 2015 Aug 13. 10.1186/s12877-015-0102-4 26268660 PMC4535784

[B26] Langa K. M. Larson E. B. Crimmins E. M. Faul J. D. Levine D. A. Kabeto M. U. (2017). A Comparison of the Prevalence of Dementia in the United States in 2000 and 2012. JAMA Intern. Med. 177, 51–58. 10.1001/jamainternmed.2016.6807 27893041 PMC5195883

[B27] Lee M. Khan M. M. (2016). Gender Differences in Cost-Related Medication Non-adherence Among Cancer Survivors. J. Cancer Surviv 10 (2), 384–393. 10.1007/s11764-015-0484-5 26350680

[B28] Matsunaga S. Kishi T. Iwata N. (2014). Combination Therapy with Cholinesterase Inhibitors and Memantine for Alzheimer's Disease: A Systematic Review and Meta-Analysis. Int. J. Neuropsychopharmacol. 18, pyu115. Published 2014 Dec 28. 10.1093/ijnp/pyu115 25548104 PMC4376554

[B29] Matthews F. E. Arthur A. Barnes L. E. Bond J. Jagger C. Robinson L. (2013). A Two-Decade Comparison of Prevalence of Dementia in Individuals Aged 65 Years and Older from Three Geographical Areas of England: Results of the Cognitive Function and Ageing Study I and II. Lancet 382, 1405–1412. 10.1016/S0140-6736(13)61570-6 23871492 PMC3906607

[B30] Matthews K. A. Xu W. Gaglioti A. H. Holt J. B. Croft J. B. Mack D. (2019). Racial and Ethnic Estimates of Alzheimer's Disease and Related Dementias in the United States (2015-2060) in Adults Aged ≥65 years. Alzheimers Dement 15, 17–24. 10.1016/j.jalz.2018.06.3063 30243772 PMC6333531

[B31] Mazure C. M. Swendsen J. (2016). Sex Differences in Alzheimer's Disease and Other Dementias. Lancet Neurol. 15, 451–452. 10.1016/S1474-4422(16)00067-3 26987699 PMC4864429

[B32] McMichael A. J. Zafeiridi E. Passmore P. Cunningham E. L. McGuinness B. (2020). Factors Associated with Mortality Including Nursing Home Transitions: A Retrospective Analysis of 25,418 People Prescribed Anti-Dementia Drugs in Northern Ireland. J. Alzheimers Dis. 73, 1233–1242. 10.3233/JAD-190751 31903992 PMC7081092

[B33] Mielke M. M. Vemuri P. Rocca W. A. (2014). Clinical Epidemiology of Alzheimer's Disease: Assessing Sex and Gender Differences. Clin. Epidemiol. 6, 37–48. Published 2014 Jan 8. 10.2147/CLEP.S37929 24470773 PMC3891487

[B34] National Committee to Preserve Social Security and Medicare (2021). Medicare and Women Economic Status and Spending for Health Care. Available online: https://www.ncpssm.org/eleanors-hope/issue-briefs/medicare-and-women-2/(Accessed Mar 6, 2021).

[B35] Nebel R. A. Aggarwal N. T. Barnes L. L. Gallagher A. Goldstein J. M. Kantarci K. (2018). Understanding the Impact of Sex and Gender in Alzheimer's Disease: A Call to Action. Alzheimers Dement 14, 1171–1183. 10.1016/j.jalz.2018.04.008 29907423 PMC6400070

[B36] O'Brien J. T. Holmes C. Jones M. Jones R. Livingston G. McKeith I. (2017). Clinical Practice with Anti-dementia Drugs: A Revised (Third) Consensus Statement from the British Association for Psychopharmacology. J. Psychopharmacol. 31, 147–168. 10.1177/0269881116680924 28103749

[B37] Owens G. M. (2018). Gender Differences in Health Care Expenditures, Resource Utilization, and Quality of Care. J. Manag. Care Pharm. 14 (Suppl. 3), 2–6. 10.18553/jmcp.2008.14.S6-A.2 PMC10438189 18439060

[B38] Rountree S. D. Chan W. Pavlik V. N. Darby E. J. Siddiqui S. Doody R. S. (2009). Persistent Treatment with Cholinesterase Inhibitors And/or Memantine Slows Clinical Progression of Alzheimer Disease. Alzheimers Res. Ther. 1, 7. 10.1186/alzrt7 19845950 PMC2874259

[B39] Scacchi R. Gambina G. Broggio E. Corbo R. M. (2014). Sex and ESR1 Genotype May Influence the Response to Treatment with Donepezil and Rivastigmine in Patients with Alzheimer's Disease. Int. J. Geriatr. Psychiatry 29, 610–615. Epub 2013 Oct 22. 10.1002/gps.4043 24150894

[B40] U.S. Food and Drug Administration (2021). U.S. Food and Drug Administration. Available online: https://www.accessdata.fda.gov/scripts/cder/daf/(Accessed August 10, 2021).

[B41] Vaidya V. Partha G. Karmakar M. (2012). Gender Differences in Utilization of Preventive Care Services in the United States. J. Womens Health (Larchmt) 21, 140–145. 10.1089/jwh.2011.2876 22081983

[B42] van de Glind E. M. van Enst W. A. van Munster B. C. Olde Rikkert M. G. Scheltens P. Scholten R. J. (2013). Pharmacological Treatment of Dementia: A Scoping Review of Systematic Reviews. Dement Geriatr. Cogn. Disord. 36, 211–228. 10.1159/000353892 23941762

[B43] Virani S. S. Woodard L. D. Ramsey D. J. Urech T. H. Akeroyd J. M. Shah T. (2015). Gender Disparities in Evidence-Based Statin Therapy in Patients with Cardiovascular Disease. Am. J. Cardiol. 115, 21–26. 10.1016/j.amjcard.2014.09.041 25456865

[B44] Wattmo C. Wallin A. K. Londos E. Minthon L. (2011). Predictors of Long-Term Cognitive Outcome in Alzheimer's Disease. Alzheimers Res. Ther. 3 (4), 23. Published 2011 Jul 20. 10.1186/alzrt85 21774798 PMC3226278

[B45] Winblad B. Jones R. W. Wirth Y. Stöffler A. Möbius H. J. (2007). Memantine in Moderate to Severe Alzheimer's Disease: A Meta-Analysis of Randomised Clinical Trials. Dement Geriatr. Cogn. Disord. 24, 20–27. 10.1159/000102568 17496417

[B46] Winblad B. Amouyel P. Andrieu S. Ballard C. Brayne C. Brodaty H. (2016). Defeating Alzheimer's Disease and Other Dementias: A Priority for European Science and Society. Lancet Neurol. 15, 455–532. 10.1016/S1474-4422(16)00062-4 26987701

[B47] Winslow B. T. Onysko M. K. Stob C. M. Hazlewood K. A. (2011). Treatment of Alzheimer Disease. Am. Fam. Physician 83 (12), 1403–1412. 21671540

[B48] Zuckerman I. H. Ryder P. T. Simoni-Wastila L. Shaffer T. Sato M. Zhao L. (2008). Racial and Ethnic Disparities in the Treatment of Dementia Among Medicare Beneficiaries. J. Gerontol. B Psychol. Sci. Soc. Sci. 63 (5), S328–S333. 10.1093/geronb/63.5.s328 18818454 PMC3781927

