# Supplementary material for: Gender Disparities in Anti-dementia Medication Use among Older Adults: Health Equity Considerations and Management of Alzheimer's Disease and Related Dementias
Source: Front Pharmacol. 2021 Aug 25;12:706762. doi: 10.3389/fphar.2021.706762 (PMC8424001; doi:10.3389/fphar.2021.706762)

Figure S1: ROC curve of the logistic regression model for beneficiaries with ADRD (N=1,240)


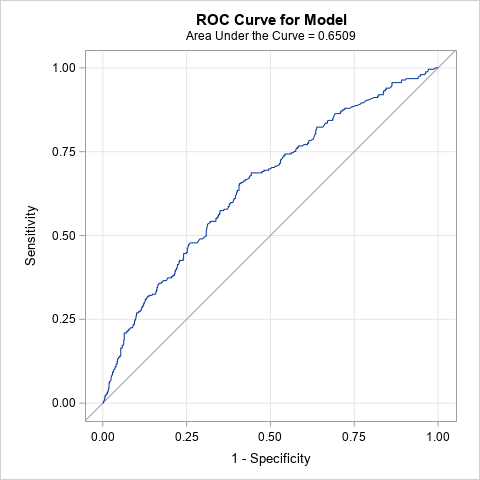


Figure S2. ROC curve of the logistic regression model for beneficiaries with AD (N=307)


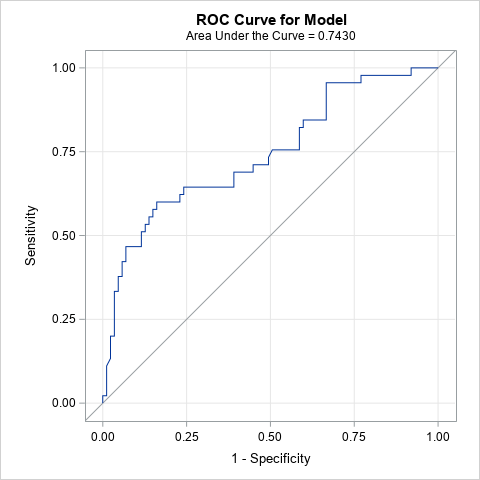


Figure S3. ROC curve of the logistic regression model for beneficiaries with AD-related dementias only (N=993)


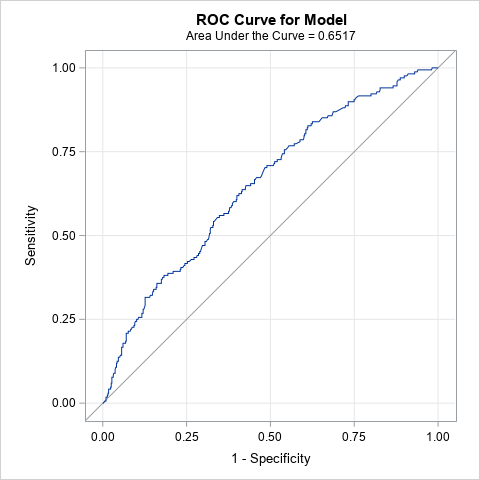

Supplement: Supplementary file 2 [file DataSheet1.docx]
